# Assessing the impact of the coronavirus pandemic on the mental health status of intensive care unit nurses: a systematic review

**DOI:** 10.1186/s12912-025-03117-6

**Published:** 2025-05-02

**Authors:** Peter Onchuru Mokaya, Nancy Ntinyari, Godfrey Limungi, Evans Kiptulon Kasmai, Hideg-Fehér Gabriella

**Affiliations:** 1 Doctoral School of Health Sciences, Faculty of Health Sciences, University of Pécs, Pecs, Hungary; 2 Faculty of Health Sciences, University of Pécs, Pecs, Hungary; 3 Faculty of Health Sciences Institute of Physiotherapy and Sport Sciences, University of Pécs, Pecs, Hungary

**Keywords:** ICU, COVID-19, Mental health, Nurses, Pandemic

## Abstract

**Introduction:**

The Coronavirus pandemic (COVID-19) was first identified in December 2019 in Wuhan, China, and later caused a severe health crisis, causing massive disruptions to most healthcare systems worldwide. During this pandemic period, the structure of the Intensive Care Unit (ICU) activities changed fast. It was observed that the mental health of ICU nurses reached levels of extreme clinical and psychological concern. This paper aims to shed light on how COVID-19 affected ICU nurses’ mental health.

**Methods:**

A literature review of articles published on this topic from January 2020 to December 2024. English-language, peer-reviewed, mixed-methods, qualitative, and quantitative research on the mental health outcomes of ICU nurses were included while studies without primary data, non-ICU nurses, and non-peer-reviewed publications were excluded. To identify relevant literature, we searched five databases, including PubMed, MEDLINE, CINAHL, Web of Science, and Embase. Additionally, grey literature sources, including Google Scholar and Research Gate, were also searched. Narrative synthesis was used to evaluate both quantitative and qualitative data.

**Results:**

A total of 23 articles were reviewed. The most prevalent mental health issues were depression, anxiety, fear, and post-traumatic stress disorder (PTSD). The effects of burnout, illness, exhaustion, physical strain, sleep disturbances, and ongoing job stress were equally detrimental to the health of ICU nurses. The nurses’ health declined as a result of the new procedures and working environment, the enormous workload, the continued exhaustion, the concerns for their families and themselves being infected by COVID-19, the social reaction, and seeing the death toll rise.

**Conclusion:**

The COVID-19 pandemic had a negative impact on nurses’ mental health well-being such as stress, depression, post-traumatic stress disorder, insomnia, anxiety, and fear. Sustainable support systems, networks and plans ought to be made available. Due to unique working conditions of ICU nurses and in readiness for similar pandemics in future, legislators should focus on the mental health of ICU nurses because they play a critical role in managing public health crises as frontline health solders.

## Background

Coronaviruses caused significant global health crises, including severe acute respiratory syndrome (SARS) in 2002–2003 and Middle East Respiratory Syndrome (MERS) in 2012 [1]. The emergence of severe acute respiratory syndrome coronavirus 2 (SARS-CoV-2) in Wuhan, China, led to the coronavirus disease 2019 (COVID-19) pandemic, officially declared by the World Health Organization on March 11, 2020 [2]. The pandemic fundamentally disrupted healthcare systems worldwide, placing unprecedented strain on healthcare professionals [3]. The unpredictable and potentially lethal nature of COVID-19, coupled with media coverage of rising infections, such as the February 2020 announcement that over 3,000 healthcare workers in China had contracted the virus, heightened fear and stress among HCPs. ICU nurses, already prone to higher levels of emotional stress pre-pandemic, faced exacerbated challenges due to increased ICU capacities, longer shifts, and the critical nature of their work [4, 5].

Mental health issues, including depression, anxiety, and post-traumatic stress disorder (PTSD), became prevalent among ICU nurses during the pandemic. Previous research highlights that ICU nurses experience higher rates of PTSD compared to their counterparts in other units, largely due to their involvement in providing critical care for the most severely ill patients [6, 7]. The psychological burden was further amplified by fears of contracting the virus, infecting loved ones, inadequate personal protective equipment (PPE), and distress over patient mortality despite best efforts [8, 9]. Burnout, defined by emotional exhaustion, depersonalization, and reduced personal accomplishment, surged during the pandemic. The proportion of nurses reporting mental health symptoms increased from 60 to 80% globally in the first wave of the pandemic [10–12]. Factors such as increased workloads, role conflict, and societal changes further contributed to the decline in nurses’ mental health [13, 14]. Burnout not only undermines nurses’ well-being but also jeopardizes patient care outcomes and the resilience of the healthcare workforce [8].

Several authors have completed reviews of the literature on nurses and the COVID-19 epidemic. As a way of example, Mobarki [15] conducted a thorough literature research to learn about the perspectives and experiences of intensive care unit nurses and how this impacted their careers. A similar comprehensive literature study was done by Fernandez et al. [16], to emphasize the experiences of acute care hospital nurses during respiratory pandemics. Yasin et al. [17], conducted a systematic review and meta-analysis to find out how the COVID-19 epidemic affects nurses’ job satisfaction, discover contributing factors, analyze the effects of job dissatisfaction, and look at ways to mitigate it. A Comprehensive Review of the Literature on the Experience of Intensive Care Unit (ICU) Nurses During COVID-19 was also done Bt. Abdul Hamid et al. [18]. All these literature review focused on nurses and COVID-19 and no review was specifically done to focus on the mental status of ICU nurses during the COVID-19 pandemic.

Systematic reviews and meta-analyses focusing on the mental health status of ICU nurses during the pandemic are limited. While prior studies have explored burnout and psychological stress among healthcare workers, there is a need for evidence synthesizing the unique challenges faced by ICU nurses during the COVID-19 outbreak. While the proportion of nurses reporting mental health symptoms increased from 60 to 80% globally in the first wave of the pandemic, we delve on the mental health status of ICU nurses in this study because of their unique working environment that is characterized by continuous exposure to severe traumatic cases, being part of end-of-life care, being subjected to their safety, safety of their patients and families during the Covid-19 pandemic. As frontline health care warriors, they form the biggest pie of the health care workforce that are subjected to post-traumatic stress experiences, burnout, and uncertainties associated with pandemics.

This study aims to examine the influence of the COVID-19 pandemic on the mental health status of intensive care unit nurses after coronavirus pandemic outbreak, providing insights into their psychological and emotional challenges. Additionally, the research explores the implications of these challenges on patient care and emphasizes the importance of early intervention to support healthcare professionals showing signs of PTSD. The findings aim to inform improved support systems, improve resilience within the nursing workforce, and better prepare healthcare systems for future pandemics.

## Materials and methods

### Study design

This study was a systematic review conducted to synthesize existing evidence on the psychological effects of the COVID-19 pandemic among ICU nurses. This design was chosen as it allows for comprehensive identification, analysis, and evaluation of relevant studies, providing a robust foundation for understanding the topic. We conducted this systematic review from October 2024 to December 2024.

### Condition or domain being studied

Intensive care unit nurses have faced unprecedented challenges, including increased workloads, high patient mortality rates, and prolonged exposure to traumatic and stressful events, which have heightened their risk of adverse mental health outcomes [4–6, 19].

The study aims to explore the impact of the pandemic on the mental well-being of this specific group of healthcare professionals, whose critical role in patient care places them at the forefront of the crisis. Understanding these mental health impacts is essential for informing interventions, policies, and support systems to promote resilience and mitigate long-term psychological effects among ICU nurses.

The World Health Organization defines mental health as, *‘a state of mental well-being that enables people to cope with the stresses of life*,* to realize their abilities*,* to learn well and work well*,* and to contribute to their communities.’* [20].

Mental disorder is defined as ‘*a syndrome characterized clinically significant disturbance in an individual’s cognition*,* emotional regulation*,* or behavior that reflects a dysfunction in the psychological*,* biological*,* or developmental processes that underlie mental and behavioral functioning*’ [20].

This study will incorporate different branches of mental illness/disorders, which include stress, depression, anxiety, post-traumatic stress disorder, burnout, and insomnia.

Stress is a state of worry or mental or emotional tension caused by a difficult situation [21]; Anxiety can be described as a normal reaction to stress while anxiety disorder can be defined as a state of mind that is typified by feelings of concern, fear, or uneasiness, frequently related to uncertain or future occurrences [22]; Depression is characterized by enduring melancholy, hopelessness, and a loss of interest or enjoyment in activities, persistent sadness, inability to carry out daily activities, low concentration and decreased energy [23, 24]; Post-traumatic stress disorder (PTSD) is a mental health condition triggered by experiencing or witnessing a traumatic event, such as violence, accidents, or natural disasters. Symptoms may also include hyper arousal, unfavorable changes in mood and thought processes, numbing and avoiding reminders of trauma [25]; Burnout is a condition of physical, emotional, and mental fatigue brought on by ongoing stress, and is frequently associated with a job or providing care. It reflects a psychological syndrome stemming from chronic emotional and interpersonal stressors in professional or caregiving contexts [26, 27]; The symptoms of insomnia include trouble getting asleep, remaining asleep, or waking up too early and not being able to go back to sleep [28].

### Registration of the research protocol

To ensure methodological transparency, we registered the research protocol with the International Prospective Register of Systematic Reviews (PROSPERO) with registration number CRD42024618004 before conducting this study, adhering to the international standards of systematic reviews. While this study involved only publicly available peer-reviewed articles, reviewers conformed with the ethics of not pre-selecting articles that could have desired results.

### Research question

Our primary research question was formulated using the Population, Exposure, Comparison, and Outcome (PECO) framework [29]. Population: ICU nurses globally, Exposure: COVID-19 Pandemic, Comparator: not applicable, Outcome: Mental health outcomes, e.g., depression, anxiety, fear, PTSD, burnout, work-related stress, physical strains, and insomnia were included. These were included because their incidence and prevalence are majorly event-based conditions. In several epidemiological studies, events have been termed as determinants of diseases and conditions. We treat COVID-19 as one of the major epidemiological events in the history of medicine [30–33]. COVID-19 was a social and epidemiological determinant of Mental health outcome among ICU nurses [34, 35]. Our study was guided by the following research question: *What has been the effect of the COVID-19 pandemic on the mental health of Intensive Care Unit nurses (ICU) globally?*

### The search strategy

A comprehensive search strategy was developed to capture a wide array of studies addressing this topic. Search terms were constructed using the keywords “COVID-19,” “Coronavirus pandemic 2019,” “ICU nurses,” “mental health,” and “Psychological impact.” Boolean operators AND/OR were utilized to ensure precision and inclusivity.

We searched five databases, including PubMed, MEDLINE, CINAHL, Web of Science, and Embase, to identify relevant literature between January 2020 and November 2024. Additional grey literature sources, including Google Scholar and Research Gate, were also searched. This included published papers and conference abstracts whose language is strictly English. We employed inclusion and exclusion criteria as per details in Table 1. The search was conducted considering the title or abstract using the following search strategy: (“ICU nurses” OR “Intensive care unit nurses” OR “Critical care nurses” OR “Healthcare workers” OR “Nurses”) AND (“COVID-19” OR “Coronavirus pandemic” OR “SARS-CoV-2” OR “COVID-19 outbreak” OR “Pandemic”) AND (“Mental health” OR “Psychological effects” OR “Psychological impact” OR “Mental health outcomes” OR “Anxiety” OR “Depression” OR “Burnout” OR “Stress” OR “PTSD” OR “Insomnia” OR “Psychological distress” OR “Well-being” OR “Emotional impact”).

**Table 1:** Inclusion and exclusion criteria

Criterion	Inclusion	Exclusion
Study type	Qualitative, Quantitative and mixed methods	Opinions, editorials, commentaries, letters and essays
Study design	Cross-sectional, longitudinal, case-control, cohort studies and mixed methods,	Case studies, systematic reviews, meta-analysis, and studies lacking primary data
Population	ICU nurses	Non-ICU nurses, mixed cadre without separate data for ICU nurses,
Exposure	ICU nurses exposed to COVID-19 environment (ICU nurses attending to COVID-19 patients)	ICU and non ICU nurses not exposed to COVID-19 environment (Nurses in non ICU departments, Nurses not attending to COVID-19 patients)
Outcome assessed	Mental health outcomes such as anxiety, depression, PTSD, burnout, stress, work-related stress and insomnia	Studies that did not explicitly assess mental health outcomes.
Language	Peer reviewed articles in English	Articles written in languages other than English
Publication type	Peer reviewed	Non-peer reviewed or articles without access

### Screening of the article

All articles retrieved through database searches were imported into EndNote for consolidation and deduplication. Subsequently, independent reviewers among NN, MO, EKK, and GML conducted a three-step screening process.

Title Screening: Titles were screened for relevance; abstract screening: Abstracts were evaluated to confirm alignment with the inclusion criteria; full-text screening: Eligible studies underwent thorough full-text analysis to finalize the selection. Discrepancies were resolved through discussion and consultation, and where consensus was not reached, a third reviewer was involved.

### Data extraction

A standardized pre-tested data extraction form agreed upon by the authors was used to systematically collect from the included studies. Data collected from the articles included the following: main author, country, year of publication, main objective/aim, study design, sample size, and the study’s main mental health outcomes.

### Quality appraisal and risk of bias of the included studies

The methodological quality of the included studies was assessed using the Mixed Methods Appraisal Tool (MMAT) [36]. The MMAT is a validated tool designed to evaluate various study designs, including qualitative, quantitative, mixed-method studies, randomized controlled trials, and non-randomized studies. The MMAT’s comprehensiveness and precision made it the ideal choice for assessing the diverse methodological studies included in our research Each study was appraised based on five methodological criteria specific to its design.

For each criterion met, a study was assigned a score of “Yes” (1 point or 20%), whereas unmet criteria were scored “No” (0 points or 0%). The total quality score was determined by summing the individual scores, resulting in an overall percentage score ranging from 0 to 100%. Studies were categorized into low quality (≤ 40%), moderate quality (41–60%), and high quality (≥ 61%). Only studies scoring from 60% were included in the final analysis.

To enhance reliability and reduce bias, two independent reviewers (NN and PM) assessed each study. Any discrepancies in scoring were discussed and resolved through consensus. If disagreements persisted, a third reviewer (EK) was consulted to reach a final decision.

### Strategy for data synthesis and analysis

To ensure that data synthesis was rigorous, credible, and transparent, we laid down a prior justification of the study with clear inclusion and exclusion criteria, clear data extraction and analysis procedure, and acknowledgment of bias that might emanate from different study designs.

In our analysis, we first thematically analyzed the quantitative findings and then mapped the qualitative results to the emerged themes, as explained below.

Due to the expected heterogeneity in study designs, populations, and mental health outcomes, the data synthesis and analysis for this review employed a narrative synthesis approach. The narrative synthesis was guided by thematic organization, grouping studies by key mental health outcomes.

Quantitative data, such as prevalence rates, mean scores, and effect sizes, was summarized descriptively and, where feasible, presented in tabular for comparison.

**Table 2:** Quality assessment of included studies

Study ID	Study design	Clear research question	Appropriate methodology	Data collection suitable	Data interpretation justified?	Conclusions supported by data	Total score (%)	Quality category
1	Qualitative	Yes (1)	Yes (1)	Yes (1)	No (o)	Yes (1)	80	High
2	Quantitative	Yes (1)	No (0)	Yes (1)	Yes (1)	No (0)	60	Moderate
3	Quantitative	Yes (1)	Yes (1)	Yes (1)	Yes (1)	Yes (1)	100	High
4	Qualitative	Yes (1)	Yes (1)	Yes (1)	Yes (1)	NO (0)	80	High
5	Quantitative	Yes (1)	Yes (1)	Yes (1)	No (0)	No (0)	60	Moderate
6	Qualitative	Yes (1)	Yes (1)	Yes (1)	Yes (1)	Yes (1)	100	High
7	Quantitative	Yes (1)	No (0)	Yes (1)	Yes (1)	No (0)	60	Moderate
8	Quantitative	Yes (1)	No (0)	Yes (1)	No (0)	Yes (1)	60	Moderate
9	Quantitative	Yes (1)	No (0)	Yes (1)	Yes (1)	Yes (1)	80	High
10	Quantitative	Yes (1)	Yes (1)	N0 (1)	No (0)	Yes (1)	60	Moderate
11	Mixed methods	Yes (1)	Yes (1)	Yes (1)	No (0)	N0 (0)	60	Moderate
12	Quantitative	Yes (1)	Yes (1)	No (0)	Yes (1)	No (0)	60	Moderate
13	Quantitative	Yes (1)	Yes (1)	Yes (1)	Yes (1)	Yes (1)	100	High
14	Quantitative	Yes (1)	Yes (1)	Yes (1)	No (0)	Yes (1)	80	High
15	Quantitative	Yes (1)	Yes (1)	Yes (1)	Yes (1)	Yes (1)	100	High
16	Quantitative	Yes (1)	Yes (1)	Yes (1)	Yes (1)	Yes (1)	100	High
17	Mixed method	Yes (1)	No (o)	No (0)	Yes (1)	Yes (1)	60	Moderate
18	Quantitative	Yes (1)	Yes (1)	Yes (1)	Yes (1)	Yes (1)	100	High
19	Qualitative	Yes (1)	Yes (1)	Yes (1)	Yes (1)	Yes (1)	100	HIgh
20	Quantitative	Yes (1)	Yes (1)	Yes (1)	No (0)	N0 (0)	60	Moderate
21	Quantitative	Yes (1)	Yes (1)	No (0)	Yes (1)	Yes (1)	80	High
22	Quantitative	Yes (1)	Yes (1)	Yes (1)	No (0)	Yes (1)	80	High
23	Quantitative	Yes (1)	Yes (1)	No (0)	Yes (1)	No (0)	60	Moderate

Table 2 gives a summary of the quality assessment findings, including the distribution of quality scores across the 23 included studies.

During data extraction and categorization, we included quantitative and qualitative studies. In conducting data extraction and categorization, we were guided by our inclusion and exclusion criteria and methodological rigor. We included all studies that highlighted the mental status of intensive care unit nurses during the COVID-19 pandemic.

Data from the screened publications was mined and charted according to study design, sample size, country, data collection methods, and a summary of the key findings. To ensure validity and reliability results from surveys, interviews and mixed methods were triangulated.

Quantitative data included the incidence rates of stress, post-traumatic stress disorder, anxiety, burnout, depression, and other mental health symptoms as well as their associated predictors. We extracted major themes from qualitative studies as they related to the mental health status of ICU nurses during the COVID-19 pandemic.

We identified and categorized data into major themes which included major mental health symptoms, depression, stress and post-traumatic stress disorder, anxiety, burnout, and sleep quality. We added emerging themes from the qualitative studies to enrich and complete our narrative synthesis and capture different experiences from different geographical locations.

During quantitative data synthesis, descriptive analysis was done based on the major themes that emerged (mental health symptoms, influential factors, and coping strategies). Comparative analysis was done to highlight the effect of COVID-19 in different waves and geographical locations, statistical outcomes were extracted and highlighted and sub group analysis done to explore how factors like work experience, age, gender, and perceptions contributed to the mental health status of ICU nurses during the COVID-19 pandemic.

Qualitative data synthesis included extraction and highlighting key themes as reported in the selected studies and used the findings to enrich the quantitative reports under each theme.

After analyzing quantitative and qualitative studies separately, a comparative analysis of the findings was done to converge and integrate quantitative and qualitative findings to give a statistical score and enriched qualitative explanation of the findings. Divergent findings concerning the variability of the different mental health challenges, geographical location, various pandemic waves were explored and incorporated in the findings.

To make this work clearer to readers’ tables and summaries of quantitative and qualitative findings were imprinted in the results and methodology sections.

We reported our findings based on the influence of the COVID-19 pandemic on the mental health status of ICU nurses and the potential implications of the identified psychological challenges on patient care and the ICU nursing profession.

## Results

**Fig. 1:**
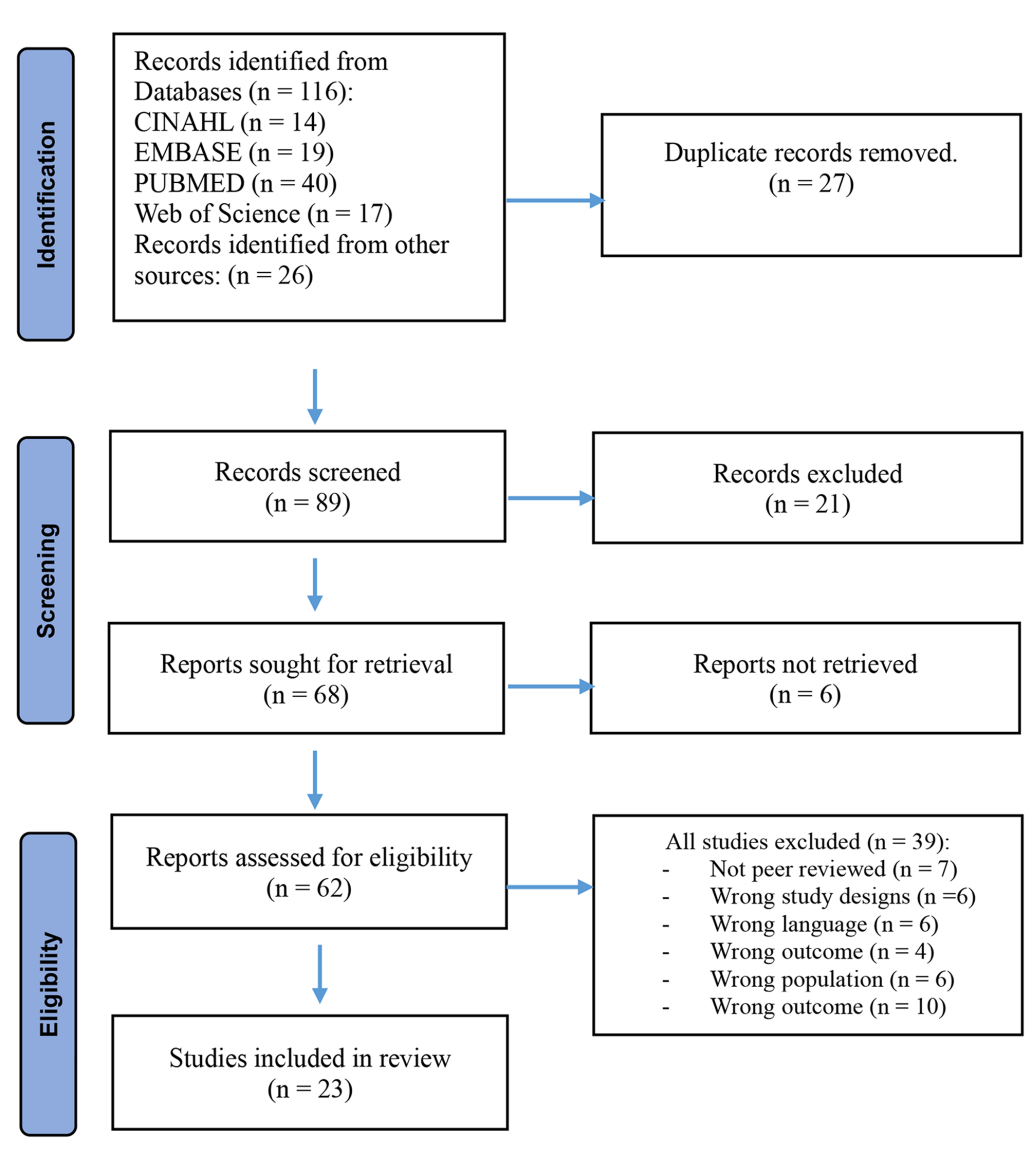
PRISMA of the search flow

As per our study on assessing the effect of the Coronavirus Pandemic on the Mental Health Status of Intensive Care Unit Nurses, a total of 116 published articles were scanned based on the inclusion and exclusion criteria. Out of the total reviewed articles finally 23 articles were obtained as outlined in Fig. 1. We reviewed 16 quantitative and correlational articles with a cross sectional design, four qualitative, and three mixed designs. Summary of findings can be found in Table 3.

### Mental health symptoms

Mental health symptoms and experiences were reported in several studies that involved the ICU nurses during the COVID-19 pandemic. A qualitative study by Peñacoba et al. [3], examined the perspectives and experiences of nurses who worked in an intensive care unit (ICU) during the COVID-19 pandemic. Results of their study showed that providing nursing care, resource management and safety, professional relationships and fellowship, and psychosocial and emotional stress were great pointers that needed to be considered when assessing and supporting nurses in the mental health frontiers.

To assess nurses’ experiences in caring for patients in intensive care units who had been diagnosed with COVID-19, a qualitative study found that, when caring for COVID-19 patients, all nurses encountered negative emotions, physical, psychological, and social challenges, which prompted them to employ coping mechanisms [37].

In the quest to be aware of the experiences ICU nurses have when caring with COVID-19 patients, a qualitative study by Conz et al. [38], pointed out that, due to the volume of care they provided during the COVID-19 epidemic, the ICU nurses made requests regarding their working conditions, professional recognition and training, and support for their physical and mental health in Brazil.

Again another study illustrated that there was a negative correlation between resilience and adverse mental health outcomes among ICU nurses during the COVID-19 pandemic [39].

To get psychological feedback from American intensive care unit (ICU) professionals regarding the challenging aspects of caring for patients with coronavirus disease, Kleinpell et al. [40], conducted a descriptive survey which reported that they had great concerns about exposing coronavirus to their family members, availability of respirators and other personal protective equipments.

In a study done by Baraka et al. [41], on predictors of ICU nurse’s stress anxiety and depression in response to the COVID-19 pandemic, it was found that the main forecaster of stress was how many coworkers tested positive for COVID-19 (*p* < 0.001) and how the hospital was equipped (*p* = 0.01). Major forecasters of nervousness were age, sex, good income (*p* < 0.001), the number of years one has worked, number of hours spent nursing COVID-19 patients (*p* = 0.04), regular training, how many coworkers tested positive (*p* = 0.01) and how the hospital was equipped (*p* = 0.02). Lastly, major forecast of hopelessness involved sex, previous physical problems (*p* = 0.04) level of education, how the hospital was equipped, previous mental problems (*p* < 0.001) and how many coworkers tested positive (*p* = 0.001). Again, a study by Jose et al. [39], found a negative relationship between resilience and poor mental outcome.

### Stress and post traumatic stress disorder

The studied ICU nurses experienced extremely severe stress that ranged from 10 to 72.3% [41, 42]. Other Incidences of stress were reported by different authors as follows, 41.7% [43], 68.5% distress [39], 38.5% severe stress [41], 38% moderate stress, 18% severe stress [44], Moderate stress (mean 10.46) [3], 54% stress [45], 22.2% PSTD [46], 50% PSTD [45], and 36.8% PTSD [47].

A cross-sectional study done by Peñacoba et al. [3], found out that there was a strong indirect influence of perceived stress levels on aspects of physical and mental health. In particular, higher resilience was linked to better physical and mental health, while lower stress perception and greater self-efficacy were associated with higher resilience (B = − 0.03; SE = 0.02; 95% confidence interval [CI] = [− 0.07, − 0.01]; B = − 0.03, SE = 0.01, 95% CI = [− 0.07, − 0.01], respectively). The link between the experience of stress and the elements of physical and mental health was found to be mediated by self-efficacy alone (B = -0.07; SE = 0.05; 95% CI = [-0.18, -0.03]; B = -0.09; SE = 0.04; 95% CI = [-0.17, -0.24], respectively). Meanwhile, resilience was not a strong mediator of these relationships.

In a quest to assess the levels of perceived stress and resilience among critical care nurses, a correlational study done by Almegewly et al. [44], found a correlation between stress and the COVID-19-related working environment. ICU nurses with a positive outlook on COVID-19 reported a relatively low stress level compared to those who were anxious about COVID-19. Although a large number of neonatal intensive care unit nurses and other healthcare workers deployed in the emergency department registered high levels of COVID-19-related stress, neonatal intensive care unit nurses with 10–15 years of work experienced stress with a significant level of p-value < 0.030.

Similarly, a study done by Heesakkers et al. [46], to find out how the first COVID-19 spike affected the mental health of critical care unit nurses and the risk factors that went along with it revealed that Symptoms of anxiety, depression, and post-traumatic stress disorder were reported by a majority of intensive care unit nurses. The first wave affected the mental health of nurses, leading to dropout jeopardy. In multivariate analysis, fear of infecting relatives (OR 2.22, CI 95% 1.61–3.39), insufficient number of personnel (OR 2.16, CI 95%, 1.43–3.26), worked in COVID-19.

**Table 3:** The summary of articles and results

No	Author reference	Geographical location	Objective	Tools of data collection	Study design	No of Cases	Outcome
1	Almegewly et al.,2022 [45]	Riyadh, Saudi Arabia	To assess the levels of perceived stress and resilience among critical care nurses.	Perceived Stress Scale of COVID-19 (PSS-10), and Connor-Davidson Resilience Scale 10 (CD-RISC-10).	Correlational cross-sectional study	139 critical care nurses	The majority of nurses experienced moderate to high levels of stress about the pandemic. There was no significant correlation between COVID-19-related stress and resilience
2	Saracoglu et al., 2020 [44]	Istanbul, Turkey	To assess the risk that a university hospital would face from the COVID-19 pandemic for medical professionals	The Fear of COVID-19 Scale, Patient Health Questionnaire, and Pittsburgh Sleep Quality Index (PSQI).	Cross-sectional survey	208 ICU healthcare providers	Nurses working in the ICU were the most vulnerable to mental health-related problems. **Depression**: 43.3% moderate, 16.3% severe; **Sleep Quality**: 45.7% poor sleep quality; **Correlation: Positive correlation between poor sleep quality and Fear of COVID-19 Scale** (*p*** < 0.005).**
3	Heesakkers et al., 2021 [44]	Netherlands	To find out how the first COVID-19 spike affected the mental health of critical care unit nurses and the risk factors.	Hospital Anxiety and Depression Scale (HADS, Impact of Event Scale-6 (IES-6, and Need for Recovery after work Scale (NFR)	Cross-sectional survey	726 ICU nurses.	Mental health was greatly impacted by the initial COVID-19 spike, and many ICU nurses were at risk of quitting and consequently endangering the continuity of treatment.
4	Crowe et al., 2021 [47]	Canada	Assess the mental health of Critical Care Registered Nurses who were directly involved in patient care.	Impact of Event Scale– Revised (IES-R), Depression, Anxiety and Stress Scale (DASS-21), Demographic Data Collection tool, Qualitative Interview guide.	Mixed method study Survey and interview	109 survey 15 interviews	The COVID-19 pandemic caused critical care nurses to undergo severe psychological discomfort. PTSD: **23% clinical concern**,** 13% probable**,** 38% significant symptoms;** Depression: **57% mild to severe;** Anxiety: **67% mild to severe;** Stress: **54% mild to severe.**
5	Bruyneel et al., 2021 [4]	French-speaking Belgium	Determine risk variables and the prevalence of burnout risk among ICU nurses.	Maslach Burnout Inventory (MBI) scale (French version)	Web-based Correlational study	1135 ICU nurses .	High rates of burnout risk among ICU nurses were attributed to high patient-to-nurse ratio, perceived increased workload, inadequate personal protective equipment, having untested COVID-19 symptoms. Burnout Risk prevalence: **68%; Depersonalization: 29%; Emotional exhaustion: 38%**
6	Heesakkers et al.,2023 [44]	Netherlands	Examine mental health following the second spike and the variables linked to indications of mental illness.	Hospital Anxiety and Depression Scale (HADS); Impact of Event Scale-6 (IES-6); Need For Recovery-11 (NFR).	Correlational	589 intensive care unit (ICU) nurses (425: cross-sectional; 164: longitudinal	**Mental Health Symptoms**: 38.2% experienced one or more symptoms; **Work-Related Fatigue**: 49.9% experienced fatigue; **Longitudinal Comparison**: Mental health symptoms remained high (33.5% vs. 38.4%), work-related fatigue increased (40.2% vs. 50.6%).
7	Saravanan et al.,2022 [5]	Greater Houston area, Texas, USA	Examining the impact of the COVID-19 on ICU nurses’ experiences with burnout and determining workable strategies for burnout prevention.	Maslach Burnout Inventory for Medical Personnel (MBI-MP); Post-traumatic Stress Disorder Checklist (PCL-5); Semi-Structured Focus Group Interviews	Mixed Method	20 registered ICU nurses	**Burnout**: High emotional exhaustion (75%), moderate depersonalization (60%), moderate personal achievement (25%). **PTSD**: 50% exhibited symptoms (Post-traumatic Stress Disorder Checklist-5 score > 33).
8	Levi et al.,2022 [51]	Southeastern United States	Examine the lived experience of ICU nurses tending to COVID-19 patients	Semi-Structured Interviews, PTSD Checklist (PCL-5), job Satisfaction Scale and Intention to Leave Job tool	Qualitative	10 ICU Nurses	Psychological strain i.e. PTSD nurses encountered when tending to COVID-19 patients and the need for policies and interventions to address the mental health and wellbeing of ICU nurses.
9	Fernández-Castillo 2021 [56]	Tertiary teaching hospital in southern Spain.	Examine and explain the perspectives and experiences of nurses who worked in an intensive care unit (ICU) during the COVID-19 pandemic.	Semi-structured interviews, Predetermined Interview Script, Video Call Recordings, Field Notes, and Transcriptions	Qualitative	102 ICU Nurses	Overwork, emotional distress, and mental anguish among ICU nurses reported.
10	Demir & Şahin 2022 [11]	Kırşehir, Turkey	To assess nurses experiences in caring for patients in intensive care units who had been diagnosed with COVID-19, the coronavirus disease.	Semi-Structured Interviews	Interviews-Qualitative	12 ICU nurses	Growing in consciousness of emotions, negative emotions, and fear of COVID-19, the main themes were: Fear and anxiety compromise care, difficulties in caring for COVID-19 patients in intensive care, and coping with the challenges in caring for COVID-19 patients in intensive care:
11	Conz et al., 2021 [38]	City of São Paulo, State of São Paulo, Brazil	Being aware of the experiences ICU nurses have when caring with COVID-19 patients.	Semi-structured phenomenological interviews conducted via digital platforms and video calls	Qualitative study	20 ICU Nurses	Led to physical wearing, which impacted the emotional sphere:
12	Peck et al.,2021 [56]	The United States of America	elucidate COVID-19’s overall effects on pediatric APRNs	Investigator-Developed Electronic Survey	Cross-sectional	886 ICU nurses affiliated with the National Association of Pediatric Nurse Practitioners	The impact of mental health on families and APRNs is among the most concerning findings; over **70%** of survey participants observed a rise in reports of mental health issues pertaining to both parents and children. **34%** of pediatric APRNs reported moderate or extreme concern for burnout; **25%** felt nervous or anxious; **15%** felt depressed or hopeless.
13	Guttormson et al., 2022 [6]	United States	to explain how COVID-19 affects the moral distress, burnout, anxiety, depression, and PTSD symptoms of ICU nurses	Measure of moral distress in healthcare professionals (MMD-HP), professional quality of life scale (PROQOL-5), trauma screening questionnaire (TSQ), patient health questionnaire anxiety and depression scale (PHQ-ADS)	Cross-sectional study	488 critical care nurses	**Burnout**: Moderate levels (84.7%); **Moral Distress**: Moderate levels; **Depression**: 44.6% moderate to severe; **Anxiety**: 31.1% moderate to severe; **PTSD**: 46.7% at risk. Anxiety, sadness, and PTSD symptoms were shown to be moderately to substantially link with both burnout and moral distress
14	Tatsuno et al., 2021 [49]	Japan	Assess the connections between nurses’ mental health and social support during the COVID-19	Multidimensional Scale of Perceived Social Support, Hospital Anxiety and Depression Scale (HADS), and Impact of Event Scale-6 (IES-6).	Cross-sectional survey	334 critical care nurses	**PTSD**: 36.8%; **Anxiety**: 47.6%; **Depression**: 56.0%; **Correlations**: Higher social support associated with lower anxiety (OR 0.979) and depression (OR 0.953); higher education level associated with lower PTSD (OR 0.622).
15	Belash et al., 2021 [19]	Tehran, Iran	Assess the level of death anxiety among ICU nurses during COVID-19	Demographic Questionnaire and Templer Death Anxiety Questionnaire:	Descriptive Cross-sectional study	110 nurses working in ICU	**Death Anxiety**: Severe anxiety in 69.1% of nurses; **Associated Factors**: Age, working hours per week, childbearing, number of patients needing end-of-life care, participation in resuscitation operations, patient death observations, satisfaction with personal protective equipment (PPE) (*p* < 0.05).
16	Kleinpell et al., 2020 [40]	United States of America	Feedback given by American intensive care unit (ICU) clinicians about the difficult parts of caring for patients with the coronavirus disease	A 16-item descriptive questionnaire	Descriptive Cross-sectional	9,120 ICU clinicians	30% of hospitalized COVID-19 patients required ICU care; the Median stress level increased from 3 (pre-pandemic) to 8 (pandemic), and among ICU nurses
17	Omidi et al.,2023 [8]	Iran University Medical Sciences in Tehran, Iran	To ascertain whether burnout and the quality of life of nurses in NICUs are related	The Maslach burnout and WHO Quality of Life-BREF tools	Correlational study	140 ICU Nurses	Emotional exhaustion (**55% low**,** 28.6% moderate**,** 16.4% hig**h), depersonalization **(56.4% low**,** 25% moderate**,** 18.6% hig**h), personal accomplishment (**79.3% high**); There is a positive correlation between personal accomplishment and all Quality of Life dimensions (*r*** = 0.40 to 0.56**), while there is a negative correlation (*r*** = − 0.47 to − 0.79**) between emotional, exhaustion and depersonalization of burnout and all QoL dimensions.
18	Sevinc et al., 2022 [55]	Istanbul, Turkey	Assess the anxiety and burnout levels of our institution’s attending physicians, residents, and nurses in the intensive care unit.	Demographic Questionnaire, Beck Anxiety Inventory (BAI), Maslach Burnout Inventory (MBI)	Cross-sectional study	104 ICU healthcare workers (25 attending physicians, 35 residents, and 43 nurses)	**Anxiety**: Moderate to severe anxiety in 44.2% of participants; **Burnout**: Emotional exhaustion (EE) median score 22, personal accomplishment (PA) median score 23, depersonalization (DP) median score 6; **Correlations**: Higher anxiety and burnout in younger healthcare workers and those tested for COVID-19; Depersonalization (DP scores and lower PA scores were observed in participants tested for COVID-19 compared to untested ones (*p*** = 0.001**,** 0.004**,** 0.004**,** respectively).**
19	Kandemir et al., 2022 [43]	Istanbul, Turkey	To evaluate ICU nurse’s levels of sleeplessness, despair, nervousness, and stress	Depression Anxiety Stress-21 Scale (DASS-21), Insomnia Severity Index (ISI), and Individual Characteristics Form	Cross-sectional study	194 ICU Nurses	**Depression**: 65.5% moderate to extremely severe; **Anxiety**: 58.3% moderate to extremely severe; **Stress**: 72.3% moderate to extremely severe; **Insomnia**: 39.7% moderate to severe; **Correlations**: Positive relationship between stress, anxiety, insomnia, and depression (*p* < 0.001).
20	Peñacoba et al., 2021 [3]	Public hospital in Madrid, Spain.	To examine how personal resources, self-efficacy, and resilience modified the relationship between stress and the physical and mental health of ICU nurses	Depression, anxiety, and stress scale in Spanish Scale (DASS-21), General self-efficacy scale (GSES), the resilience scale (RS-14), Quality-of-life components related to physical and mental health tools- mental health component (MCS), and the health-related quality-of-life scale (SF-36)	Cross-sectional study	308 intensive care nurses	**Stress**: Mean score 10.46 (SD = 4.31); **Self-Efficacy**: Mean score 29.65 (SD = 3.69); **Resilience**: Mean score 78.12 (SD = 15.31); **Quality of Life**: Physical health component (PCS) mean score 87.53 (SD = 16.51), mental health component (MCS) mean score 53.98 (SD = 22.06); **Correlations**: Stress negatively correlated with resilience (*r* = -0.31), self-efficacy (*r* = -0.21), PCS (*r* = -0.29), and MCS (*r* = -0.67).
21	Petrișor et al., 2021 [9]	Romania	Assessing moral pain and understanding the frequency of nervousness and hopelessness among ICU nurses	Measure of Moral Distress for Healthcare Professionals (MMD-HP) Score and Patient Health Questionnaire for Anxiety and Depression (PHQ-4) Score.	Cross-sectional study	79 ICU Nurses	**Moral Distress**: Mean score 106.63 (SD = 59.35); **Anxiety**: 13.92% of nurses; **Depression**: 2.53% of nurses; **Intention to Leave**: 26.58% of nurses during the pandemic; **Correlations**: Moral distress weakly correlated with anxiety and depression (*r* = 0.41).
22	Baraka et al., 2023 [41]	Alexandria, Egypt	To elucidate the factors that have a relationship with hopelessness, nervousness, and stress	Demographic and Work-Related Questionnaire, and Depression, Anxiety, and Stress Scale (DASS-21):	Cross-sectional study	200 200 critical care nurses (CCNs) from 5 ICUs in 5 hospitals	**Stress**: 38.5% severe, 10% extremely severe; **Anxiety**: 62% severe; **Depression**: 34.5% moderate; **Predictors**: Lack of hospital resources, number of infected colleagues, female gender, unsatisfactory income, and history of psychological problems.
23	Jose et al., 2022 [39]	Chandigarh, India	To assess the impact of COVID-19 patients in ICU and the psychological resilience of nurses and mental outcomes of stress, worry, fear, and sleeplessness	Perceived Stress Scale (PSS-10), Generalized Anxiety Disorder Scale (GAD-7), Fear Scale for Healthcare Professionals regarding COVID-19 Pandemic, Insomnia Severity Index, Connor-Davidson Resilience Scale-10 (CD-RISC).	Cross-sectional survey	137 ICUs nurses at a tertiary care center	Significant mental health problems of stress, worry, and sleeplessness amongst ICU nurses **Stress**: 68.5% moderate to severe; **Anxiety**: 54.7% mild to severe; **Fear**: 44% moderate to severe; **Insomnia**: 31% sub-threshold to moderate; **Resilience**: Mean score 31.23 (SD = 4.68), negatively correlated with stress (*r* = -0.45), anxiety (*r* = -0.41), and fear (*r* = -0.23).

ICU (OR 3.62, CI 95%: 1.04–12.57) and inadequate support supervisor (OR 1.80, CI 95%: 1.20–2.69) was associated with poor mental health outcome of ICU nurses. Not enough coworkers, fear of spreading the disease to family members, and working in an academic hospital were linked to higher mental symptoms. On the other hand, vacationing was linked to fewer depressive symptoms and less rehabilitation needed.

In the mixed study done by Crowe et al. [45], the participants in the questionnaires reported mild to severe depression (57%), anxiety (67%), stress (54%), and clinical concern for (23%), probable (13%) and substantial (38%) symptoms of post-traumatic stress disorder. In the interviews, psychological distress was characterized using terms such as anxiety, concern, and distress. The anxiety centered around rapidly changing knowledge and policies, communication that was both confusing and overwhelming, the challenge of addressing patient care demands in unconventional ways while ensuring safety, and the simultaneous management of personal and household obligations to oneself and one’s family.

In Examining the impact of COVID-19 on ICU nurses’ experiences with burnout and determining workable strategies for burnout prevention Saravanan et al. [5] did a mixed-method study that revealed that ten of twenty ICU nurses exhibited symptoms of post-traumatic stress disorder. Other symptoms included emotional exhaustion and depersonalization. It was found that personal, patient needs, work environment, and societal expectations were the major sources of mental health disturbances.

To examine the lived experience of ICU nurses tending to COVID-19 patients a study by Levi and Moss [48] revealed that the stressful environment that was characterized by unsatisfied support of ICU nurses from coworkers, managers, health care system were the main sources of Post-traumatic stress disorder during the COVID-19 pandemic.

In another cross-sectional study done by Tatsuno et al. [47], the mean age of the 334 responses was 37.4, and 64.4% of the respondents were female. 269 (80.5%) of the total were caring for COVID-19 patients at the time the study was carried out. The study revealed that those experiencing symptoms of post-traumatic stress disorder (PTSD) were older (*P* < 0.05), and those possessing a 4-year college degree or above had fewer PTSD symptoms (*P* < 0.05). Social support scores were lower for those who reported feeling anxious and depressed. A four-year college degree or more was substantially linked to a lower risk of developing post-traumatic stress disorder (OR 0.622, 95% CI 0.39–0.99). There was no correlation found between PTSD and social support ratings or female sex. Being female and having less social support were found to be independently related to a higher chance of anxiety symptoms. Lower social support was found to be independently linked to a higher chance of depression symptoms (OR 0.953, 95% CI 0.93–0.97).

### Sleep quality

Incidence of insomnia was reported by different authors ranged from 31 to 45.7% among ICU nurses as follows, 31% [39], 39.9% [42] and, 45.7% [49].

A cross-sectional study done by Kandemir et al. [42], to evaluate ICU nurse’s levels of sleeplessness, despair, nervousness, and stress employed a structural model equation and concluded that anxiety and insomnia scored a statistically significant (*p* < 0.001) effect on depression among ICU nurses during the COVID-19 pandemic.

A cross-sectional survey done by Saracoglu et al. [49], that sought to assess the risk that a university hospital would face from the COVID-19 pandemic for medical professionals found that depressed symptoms were considerably more common among doctors and nurses in intensive care units (*p* = 0.018). ICU nurses and doctors had considerably higher Pittsburgh Sleep Quality Index (PSQI) scores than their hospital ward colleagues (7.02 ± 4.59 vs. 4.81 ± 3.57 respectively, *p* = 0.001). For every patient, there was a significant positive connection (*p* < 0.005) between the PSQI and the overall score on the Fear of COVID-19 Scale.

To assess the impact of COVID-19 patients in ICU and the psychological resilience of nurses and mental outcomes of stress, worry, fear, and sleeplessness Jose et al. [39], did a cross-sectional survey in North India and found that ICU nurses reported high cases of anxiety, distress, fear, and insomnia.

### Depression

The incidence of depression was as low as 18.9% to as high as 68.5% among the participating ICU nurses [46, 47]. Again, other studies reported scores that were within the highlighted range as follows: 56.0% [39], 34.5% [41], 65.5% [42], 43.3% moderate, 16.3% severe [49], and 57% [45].

The works of Saracoglu et al. [49], indicated that depressive symptoms were higher in ICU nurses compared to their healthcare worker counterparts who were working in different departments. This concurred with the study of Crowe et al. [45], who concluded that mild to severe depression (57%) affected critical care registered in Canada. Similar finding were given by the study of Heesakkers et al. [46], which pointed out that 21.1% of ICU nurses experienced symptoms of depression among Dutch ICU nurses of which more than half of ICU nurses experienced at least one form of mental health problems.

The scientific work of Kandemir et al. [42], in Istanbul indicated that moderate to severe depression affected nurses who were working in the ICU and the dependent variable, depression, was found to be statistically significantly impacted by stress, anxiety, and insomnia (*p* < 0.001). Comparable results were given by the study of Tatsuno et al. [47], who found that depression symptoms, lower social support were independently associated with a higher probability (OR 0.953, 95% CI 0.93–0.97) among ICU nurses who were tackling COVID-19 pandemic in Japan.

### Anxiety

Out of the 23 studies, the prevalence of anxiety ranged from 26.2 to 67% [45, 46]. Other studies registered incidence that was within the said range as follows, 27.0% [43], 47.6% [47], 54.7% [39], 62% [41],13.92% [9], 44.2% [50], 58.3% [42], and 62% [41].

A study done by Saracoglu et al. [49], revealed that there was a significant statistical relationship between PSQI and the fear of COVID-19 among ICU nurses in Turkey. In the same note critical care registered nurses who were directly involved in patient care during the COVID-19 pandemic registered 67% of symptoms of anxiety in Canada [45]. Similarly, in Istanbul, the study of Kandemir et al. [42], showed that 58.3% of ICU nurses experienced anxiety during the COVID-19 pandemic. Stress, anxiety, and sleeplessness were found to have statistically significant effects on depression (*p* < 0.001). A study done by Heesakkers et al. [46], in the Netherlands revealed that anxiety and other mental health symptoms were linked to ICU nurses who were working in an academic hospital that responded to COVID-19 (adjusted odds ratio [aOR], 1.54; 95% confidence interval [CI], 1.02 to 2.34; *p* = 0.04).

A study that found that 54.7% of ICU nurses suffered symptoms of anxiety in North India established that with a mean percentage score of 77.5 (31.23 ± 4.68), frontline nurses’ resilience was moderate to high, and there was a negative connection between resilience and unfavorable mental outcomes [39].

A cross-sectional study done in Iran by Belash et al. [19], to assess the level of death anxiety among ICU nurses during COVID-19, the authors demonstrated that age, weekly working hours, childbearing, the number of patients requiring end-of-life care, direct involvement in resuscitation procedures, patient death observations, and satisfaction with personal protective equipment are all related to the degree of death anxiety experienced by nurses working in COVID-19 intensive care units (*P* < 005).

According to Peck and Sonney [51] a total of 796 Respondents reported negative effects in the areas of clinical, educational, personal, and research focus. The most concerning results showed that 34% of respondents expressed moderate to severe anxiety about feeling professionally burned out, 25% reported feeling apprehensive or frightened, and 15% reported feeling hopeless or sad. 40% of the ICU nurses made their own Personal Protective Equipment or re-used them, of which 22% of them felt insecure.

### Burnout

A web-based survey of 1135 nurses in French-speaking Belgium done by Bruyneel et al. [4], investigated the prevalence of burnout risk and factors associated with it among ICU nurses. The results indicated an overall burnout risk prevalence of 68%. Specifically, 29% of ICU nurses were at risk of depersonalization (DP), 31% exhibited lower personal accomplishment (PA), and 33% experienced emotional exhaustion (EE). The nurse-to-patient ratio increased to 1:3, with an elevated risk of both DP (OR = 1.38, 95%CI: 1.09–2.40) and EE (OR = 1.77, 95% CI: 1.07–2.95). Higher perceived workload during the COVID-19 pandemic correlated with an increased likelihood of all aspects of burnout. Additionally, nurses who reported COVID-19 symptoms without being tested were more likely to experience EE (OR = 1.40, 95% CI: 1.68–1.87), and the absence of personal protective equipment heightened the risk of EE (OR = 1.78, 95% CI: 1.35–3.34). Similar results were reported by Heesakkers et al. [43], who reported that 49.9% of ICU nurses faced work-related fatigue following the second spike of COVID-19 in the Netherlands.

Another study done by Saravanan et al. [5], in Texas, USA, to explore contributors to and strategies for mitigating burnout during the COVID-19 pandemic. The findings revealed that participants reported moderate depersonalization (M 9.75, SD 7.10), moderate personal achievement (M 32.05, SD 7.59), and significant emotional weariness (Mean score 32.35, SD 10.66 on the Maslach Burnout Inventory for Medical Personnel-MBI-MP). Additionally, 10 out of 20 subjects exhibited symptoms indicative of post-traumatic stress disorder (Post-traumatic Stress Disorder Checklist-PCL-5 score > 33). The study identified five main themes contributing to nurse burnout during the pandemic: personal, patient-related, co-worker-related, organizational, and societal. These themes encompassed subthemes such as emotional detachment from patients, the continuous need to justify actions to patients’ families, staff, and resource shortages, and the polarization of COVID-19 and vaccination. Participants also shared practical strategies to address burnout, including providing mental health services and public education initiatives emphasizing the gravity of the pandemic and the importance of vaccination.

Part of the study conducted by Guttormson et al. [6] in the USA found that ICU nurses reported moderate to high moral distress and burnout. Burnout and moral distress were great risks that would drive nurses from leaving the ICU profession or practice with the United States of America’s territory.

In the same note Levi and Moss [48] found out that as the ICU nurses responded to the COVID-19 crisis they suffered a great deal of post-traumatic stress disorder and this led to burnout, job dissatisfaction, and intention to quit the ICU profession. Similar results were reported by Petrișor et al. [52], where morals distress and other system factors were key drivers of nurse’s intention to quit working in the ICU department in Romania. Similarly, Sevinc et al. [50], showed that younger healthcare workers, namely nurses, and residents, who had been tested for COVID-19 had greater levels of anxiety and burnout in Turkey. In the said study, depersonalization, personal accomplishment, and emotional weariness scores varied according to the healthcare workers’ positions (*p* = 0.034, 0.001, and 0.004, respectively).

According to a correlational study done by Omidi et al. [8], 140 nurses who worked in the Neonatal Intensive Care unit nurses in Tehran during the COVID-19 pandemic period found that All quality of life measures show a negative correlation (*r* = − 0.47 to − 0.79) with emotional tiredness and depersonalization of burnout, and a positive correlation (*r* = 0.40 to 0.56) with personal success. In addition, married nurses and those with bachelor’s degrees had significantly lower emotional tiredness of *P* = 0.001 and *P* = 0.006, respectively. However, the Pearson correlation test revealed that emotional tiredness declined as marriage length *p* = 0.023 and the number of kids *p* = 0.002 increased. Moreover, emotional tiredness rises in tandem with an increase in overtime hours (*P* = 0.017). The results of the linear regression analyses showed that the only significant variable in the model (B = -0.41) was the length of marriage. This indicates that emotional tiredness reduces by 0.41 for every year that marriage is extended. It is important to acknowledge that the model’s coefficient of determination was a very tiny R2 = 0.083.

Similarly, the works of Peck and Sonney [51] showed that 34% of pediatric advanced practiced registered ICU nurses expressed moderate to severe anxiety about feeling professionally burned out during COVID-19 in the USA.

### Influential factors and predictors

From the reviewed studies, the most influential factors in the prevalence of various mental health challenges among ICU nurses were highlighted. Lack of supportive working conditions and unclear communications from the supervisor and administration contributed to the poor mental health outcomes [3, 6, 9, 19, 38, 45, 48, 53]. Most studies indicated that high workload was the major predictor of mental health challenges [3, 4, 8, 38, 43, 51]. The female ICU nurses consistently exhibited symptoms of mental illness [3, 44, 45]. Inadequate medical supplies, PPEs, and related resources influenced nurses’ mental strains as per different reviewed scientific works [4, 6, 40, 41]. Fear of COVID-19 cross-infection from relatives and colleagues predicted the mental health situation of ICU nurses [41, 46, 49, 54]. Higher ages were associated with fewer mental health challenges [54], and education level played a role in predicting poor mental health symptoms [47].

Other influential factors were, insomnia [42], work experience [43, 44], lack of leave [43], fatigue [8, 42], resilience, rapid policy changes [45], living conditions [8, 49], personal commitment [45] social support [8, 47], job security [8], self-efficacy [3] and involvement in end-of-life care [19, 39].

## Discussion

These research findings show the profound impact of the COVID-19 pandemic on the mental well-being of intensive care unit nurses compared to their counterparts in different departments. Our results conform with the works of Wozniak et al. [55], who found that ICU nurses had more symptoms of anxiety, depression, and lower well-being compared to non-ICU nurses during the COVID-19 pandemic.

In our reviews, there were higher depression rates among ICU nurses, 43.3% [49], 57% [45], compared to non-ICU nurses, 18.6% [53], 54.7% [39]. Anxiety was more profound in ICU nurses, where it was reported with a high of 67% [45], 58.3% [42], compared to non-ICU nurses who reported moderate to high 48.8% [54], 54.7% [39]. Burnout was severe in ICU nurses, where 68% of them were at risk [4], while their counterparts in other departments reported 34% risk of burnout [51]. Post-traumatic stress disorder was more pronounced in ICU nurses, where 22–23% reached a clinical concern [45, 53] compared to their counterparts in different departments that registered present but generally lower symptoms [53]. When assessing resilience, there was a weaker correlation with stress in ICU nurses [44, 51] compared to non-ICU nurses who registered a higher resilience score of 77.5% [39].

The first wave yielded symptoms of depression, PTSD, anxiety, and job burnout in a significant proportion of nurses, with women reporting higher scores than men. Stress levels were 2.5 times higher than before the pandemic and remained elevated after the surge. Inadequate ICU nurses were an obstacle as standard ICU beds could not be expanded [3, 44, 45]. Similar themes also featured in the qualitative studies that were reviewed [11, 38, 56]. This led to some ICU nurses desiring to quit their profession due to fear and an unconducive work environment [46]. Monitoring mental well-being was crucial for interventions, as persistent work stress increased the risk of mental health problems.

Lessons learned from these scientific works point out that the mental health of healthcare workers need not be overlooked especially those who are deployed in traumatizing environments like ICU and casualty. As per our evidence, healthcare institutions may lose their healthcare providers if they don’t invest in their mental health and well-being.

The impact persisted after the second surge, with ICU nurses experiencing sustained high levels of mental health symptoms and an increased likelihood of work-related fatigue. Various studies quantitative and qualitative studies highlighted burnout, worry, and increased workload, emotional toll, poor patient-family relationships, loneliness, overwhelming public response, and job satisfaction as major challenges [4–6, 43, 45, 48, 51, 56].

This is a sign of a shortage of healthcare workers worldwide and if no deliberate efforts are not made to reduce the healthcare workers-to-patient ratio, respond to mental health challenges, and offer psychosocial support to families a similar pandemic may get several countries unprepared.

ICU nurses faced difficulties in providing standard care due to evolving hospital regulations and increased workload. The use of personal protective equipment hampered therapeutic contact between nurses and patients [56, 57]. This situation was similar in most countries, especially at the beginning of the pandemic. It is assumed that most countries learnt from this and may be well prepared should a similar pandemic arises.

Nurses reported support from friends and family as crucial in overcoming challenges [37]. Pediatric nurses faced the loss of loved ones, and mental health issues rose among both parents and children. Burnout, depression, anxiety, and PTSD risk affected critical care nurses, with interrelated issues exacerbating the challenges. ICU nurses also experienced death anxiety when dealing with COVID-19 cases [3, 44, 45]. One of the reviewed qualitative research studies emphasized the necessity of professional recognition, training requirements, support, better working conditions, and adjustment to new care protocols [58].

Studies revealed varying degrees of depression, anxiety, and stress among healthcare workers, with factors such as COVID-19 testing status influencing burnout and anxiety [6, 47, 50]. Nurses reported moderate-to-severe insomnia, and studies emphasized the indirect impact of resilience and self-efficacy on perceived stress and quality of life. Moral distress during the pandemic was linked to depression, anxiety, and the desire to quit among ICU nurses.

The need for ongoing enhancement of ICU resources was highlighted, as well as the importance of resilience, particularly among nurses with more work experience [3]. Significant psychological discomfort and the necessity of early detection and all-encompassing assistance for nurses were also highlighted by the qualitative themes [11, 45].

The topics of patient isolation, increased workload, dehumanizing care, family care, specialized care, fear and emotional instability, resource management, and professional relationships were highlighted in the qualitative research. PPE, anxiety, frustration, false information, and the requirement for specialized training were among the difficulties nurses faced [56].

The studies place a strong emphasis on maximizing available resources, offering psychological assistance, and putting in place superior emergency measures. Overall, the research underscores the significant and lasting mental health challenges faced by ICU nurses during the COVID-19 pandemic.

### Study limitations

We can’t have a conclusive causal effect of COVID-19 on the mental health of ICU nurses, as most reviewed studies are cross-sectional, which means that data were collected at a single point in time. This limits us from concluding whether only COVID-19 pandemic-related events or other factors caused the observed outcomes of the mental health status of ICU nurses.

Reviewed studies used different tools to collect data for the same outcome, hence, there might be differences in how symptoms of mental health disorders were captured, therefore compromising the generalization of the results. This may make it challenging to compare results from the included studies due to the stated variability, eventually compromising the findings’ consistency and reliability.

Although most studies were of the cross-sectional design, researchers used different analytical approaches that might affect the reported descriptive and correlational degrees. Different analytical approaches may challenge us to compare results from different studies. Although most studies included different control variables, bias and confounding factors may have affected the findings of some of the included studies.

These findings are drawn from different populations, different geographical locations, different cultural contexts depending on the magnitude of the COVID-19 pandemic, health care systems, protective environment and the like. Consequently, caution should be taken while evaluating the findings and making inferences.

### Recommendation, practice and policy implications

The recommendation from the study is to monitor the risk of burnout, depression, post-traumatic stress disorder, insomnia and anxiety in ICU nurses and implement interventions to prevent and manage them. The study identifies contributors to mental health problems in ICU nurses and suggests practical mitigation methods through a participatory approach. This information can be used to design effective interventions to address pandemic-related mental health problems among ICU nurses. Additionally, to reduce or avoid PTSD in ICU nursing staff, hospital administrators, nurse managers, and occupational health nurses should implement evidence-based interventions and policies. Continuous psychological support, affordable access to mental health screenings, and maintaining privacy for PTSD symptoms diagnosis are emphasized. The study also underscores the importance of reviewing and improving policies related to pandemics to protect employee rights and ensure a safe work environment for healthcare workers.

Practice and policy implications emanating from this study point out that,

- Adequate support and a good communication system should be enhanced by the supervisors and general health care management team for ICU nurses during pandemics and other crises, since the nature of their work subjects them to the disturbance of their mental health.
- Establish a confidential psychological 24/7 support hotline for nurses and other healthcare workers who are exposed to the ICU during pandemics and related emergencies.
- Organize debrief sessions where ICU nurses express their emotions to their peers and unbiased senior colleagues in the profession in a supportive environment.
- Global, regional, and local standard operating procedures should be put in place to assess and promptly address the mental health challenges of ICU nurses regularly and be heightened during pandemics and similar crises in different cultural contexts.
- Evidence shows that the prevalence of Stress, post-traumatic stress disorders, anxiety, depression, burnout, and insomnia are high among ICU nurses hence, there should be a deliberate effort of developing preventive, curative and rehabilitative measures that targets nurses and other health care workers that works in the ICU during pandemics and beyond.
- To respond to pandemics and emergencies Ministry of Health and healthcare institutions need to develop an emergency plan that encompasses all the building blocks of health by enhancing emergency services delivery, retraining inter and intradepartmental workforce, availing essential medicines and supplies, adequate financing, and clear policy communication through strong leadership. This will cushion the mental health of all healthcare workers.
- Nursing needs to be recognized as a highly stressful profession hence resilience and social support programs should be developed to support ICU nurses in addressing their work-related adversities.
- Since females are more prone to anxiety and junior nurses are more likely to suffer from mental disorders, gender-specific intervention and assigning workload to nurses based on their experience and ability will contribute to better mental health and well-being of nurses.
- Governments should guarantee sufficient resources, such as stockpiled essential PPEs to maintain a 90-day supply, ensuring healthcare workers have the necessary resources during pandemics and other emergencies.
- Educate additional nurses to lower the patient-to-nurse ratio to prevent mental health challenges associated with heavy workloads during pandemics and clearly define nurse-to-patient ratios, such as the 1:2 ratio suggested by the American Association of Critical-Care Nurses (AACN), to guarantee proper staffing and the best possible patient care.
- Develop and deliver structured offline and online resilience programs that will capacitate health care within the critical care environment with coping skills necessary for maintaining well-being during pandemics and emergencies.
- Holiday and off days contribute to the betterment of the mental health status of ICU nurses and hence should be embraced.

## Conclusion

Mental health challenge is a reality among ICU nurses during pandemics and emergencies, and deliberate efforts are required to diagnose and promptly respond to them within a comprehensive system. Effective communication and robust emergency preparedness plans are necessary to ensure the well-being of ICU nurses and other healthcare colleagues to sustain effective healthcare delivery during crises.

## Data Availability

The authors are ready to provide the data using the systematic research protocol that was registered with PROSPERO.
